# Influence of Zwitterionic CAPB on Flocculation of the Aqueous Cationic Guar Gum/Glauconite Suspensions at Various pH

**DOI:** 10.3390/ijms222212157

**Published:** 2021-11-10

**Authors:** Ewelina Godek, Elżbieta Grządka, Urszula Maciołek, Anna Bastrzyk

**Affiliations:** 1 Department of Radiochemistry and Environmental Chemistry, Faculty of Chemistry, Institute of Chemical Sciences, Maria Curie-Skłodowska University, M. Skłodowskiej-Curie 3 Sq., 20-031 Lublin, Poland; ewelina.godek@poczta.umcs.lublin.pl (E.G.); egrzadka@poczta.umcs.lublin.pl (E.G.); 2 Analytical Laboratory, Faculty of Chemistry, Institute of Chemical Sciences, Maria Curie-Skłodowska University, M. Skłodowskiej-Curie 3 Sq., 20-031 Lublin, Poland; urszula.maciolek@poczta.umcs.lublin.pl; 3 Department of Process Engineering and Technology of Polymer and Carbon Materials, Faculty of Chemistry, Wroclaw University of Science and Technology, C.K. Norwida 4/6 Sq., 50-373 Wroclaw, Poland

**Keywords:** glauconite, cationic guar gum, CAPB, flocculation, adsorption, pH

## Abstract

The influence of the pseudoamphoteric zwitterionic surfactant cocamidopropylbetaine (CAPB) on the stabilizing flocculating properties of the aqueous suspensions of glauconite (GT) with cationic guar gum (CGG) at various pH values was investigated. The following techniques were used: turbidimetry, UV-VIS spectrophotometry, tensiometry, electrophoretic mobility measurements, SEM, CHN, XRD, and FT-IR. It was established that CGG is an effective glauconite flocculant. Moreover, the most probable mechanism that is responsible for flocculation is bridge flocculation resulting from polymer adsorption on the glauconite surface. The adsorption process is caused by electrostatic interactions between the negatively charged glauconite surface and the positively charged polymer. The amount of CGG adsorption increases with the increase of the pH, which was confirmed by the adsorption and zeta potential measurements. The addition of CAPB increases the amount of the polymer adsorption due to the formation of intermolecular polymer–surfactant complexes; however, it reduces flocculation effectiveness.

## 1. Introduction

Glauconite (GT) is a green clay mineral with the following chemical formula: (K,Na,Ca)(Fe,Al,Mg)_2_[(OH)_2_/(Al,Si)_4_O_10_] × nH_2_O [1,2]. It is a hydrated iron-rich mica clay, a mineral related to T-O-T layered illites, that usually forms in marine sediments [3,4]. Due to its large potassium content, glauconite is called an agro-mineral, and it is often used as a nutrient fertilizer for soil and is critical for the plant growth and crop development [5,6]. This mineral shows a relatively low cation exchange capacity for K^+^ ions. This exchange mainly takes place at the crystal boundary surfaces. The production of potassium fertilizers is based on the chemical treatment, volatilization at high temperature, calcination or leaching, and the preparation of salts. Treatment the strong mineral acids (HCl, HNO_3_, and H_2_SO_4_) replaces the exchangeable K^+^, Na^+^, and Ca^2+^ cations with H^+^ and partially extracts Al, Fe, and Mg [7]. Unfortunately, the described processes have complicated steps. Therefore, the use of chemically activated glauconite in a planetary mill is a “green alternative” that can be used to overcome the above-mentioned limitations and to additionally obtain a beneficial effect on the soil texture and longer retention at a lower cost [7,8]. Glauconite is also used in the process of removing heavy metal contaminants such as Pb, Cd, and Zn from water [9,10]. Many techniques can be used in this process, but flocculation and adsorption deserve special mention. Adsorption seems to be a particularly great solution because this process is quite flexible and is not overly complicated. The treated wastewater is of high quality, and the adsorbents can be regenerated by the desorption process, which is very important from the ecological point of view. Therefore, it is possible for glauconite to be used as a natural and cheap adsorbent that is useful in water treatment. Research shows that through ion exchange combined with adsorption, natural glauconite can be used to remove ammonia from contaminated waters and soils. Without pretreatment, about 20% of ammonia can be removed over the entire pH range. On the other hand, the ammonia removal capacity of glauconite treated with NaOH and NaCl increases to 88%. In the case of Zn, Cd, and Pb ions, the adsorption isotherms show that the adsorption amount increases when the pH increases; thus, the extent that water can be purified from these heavy metals also increases [11].

Cationic guar gum (CGG) is a modified guar gum that is obtained from the seeds of *Guar Cyampopis tetragonolobus* [12]. This biopolysaccharide has thickening and viscosity enhancing properties, and when mixed with surfactants, it increases their foaming properties [13]. For this reason, it is widely used in the cosmetics industry for the production of shampoos, creams, and shower gels [14,15]. The effect of cationic guar gum (CGG) and oleic acid (OA) on the emulsion stability of subtilisin-natural hydrolyzed plasma (NP) was investigated [16]. The results showed that enzyme-hydrolyzed plasma (EP) contributed to a higher ζ potential and lower initial droplet interfacial tension than NP, which improved emulsion stability. The influence of cationic guar gum on the flotation separation of galena from sphalerite was also investigated [17,18]. Diethyldithiocarbamate (DTC) acted as a galena collector, and the order in which the reagents were added had a significant effect on the separation efficiency. The results show that the addition of CGG after the DTC increases the collector adsorption on the galena surface compared to the addition of CGG before DTC, while for sphalerite, the addition order is not important, as the results showed poor collector coverage on its surface. Cationic guar gum, together with chitosan, is also used for the synthesis of various types of hydrogels to remove phosphate from water [19], with polyacrylic acid being used for biomedical and drug release applications [20,21] and are also used with stearyl methacrylate as self-regenerating flexible sensors [22]. CGG can also be a component of the hydroxyethyl cellulose composite films that are often used in the production of intelligent antibacterial packaging [23].

Cocamidopropylbetaine (CAPB) is a pseudoamphoteric surfactant with the chemical formula C_19_H_38_N_2_O_3_ [24]. This zwitterionic compound is composed of both a carboxylate anion and a quaternary ammonium cation. However, nitrogen always has a net positive charge, and thus, the molecule can never (even at high pH) become anionic. As a result, betaines do not exhibit anionic properties in alkaline solutions [25,26], meaning that is CAPB is not able to become a true amphoteric surfactant. Compared to most anionic and some non-ionic surfactants, betaine surfactants are very gentle for the skin and mucous membranes, and they also reduce irritation; as a result of this, they have found many applications in the cosmetic industry for the production of shampoos, conditioners, body washes, and other skin care products [27,28]. In addition to its many applications in cosmetology, CAPB also affects the structural, optical, morphological, and electrical properties of PbS chalcogenides [27]. Research has shown that the addition of CAPB to the PbS nanostructures in the deposition process on a glass substrate along with an increase in the surfactant concentration reduces surface roughness and improves optical properties. These features indicate that this compound can be used as an absorbing material in the production of solar cell devices. 

The literature data report no information on the influence of CGG and CAPB on the adsorption, electrokinetic, and stabilization-flocculation properties of aqueous glauconite suspensions. There is also not much information as to how pH influences these properties. Glauconite is a mineral substance whose applications can be very limited, so it is worth studying this subject, as it has great application potential in the important processes such as water purification for care skin products and for the separation of these compounds from water. Thus, the aim of the presented studies was to analyze the influence of zwitterionic, pseudoamphoteric surfactant cocamidopropylbetaine (CAPB) on the stabilizing-flocculating properties of aqueous suspensions of glauconite (GT) with cationic guar gum (CGG) at various pH values.

## 2. Results and Discussion

### 2.1. Stability Measurement

**Figure 1:**
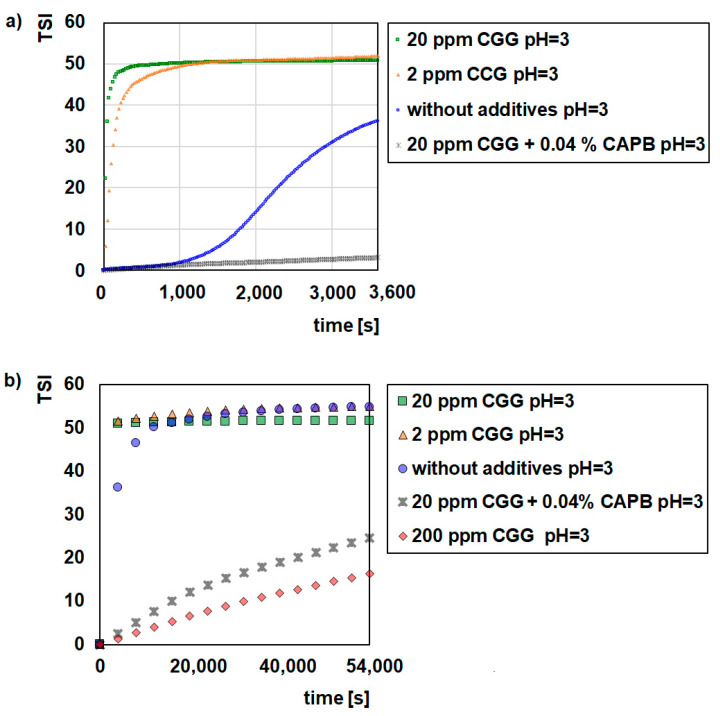
Influence of CGG with the addition of 0.04% CAPB on stability of the glauconite suspensions at pH = 3; 1 h (**a**), 15 h (**b**).

**Figure 2:**
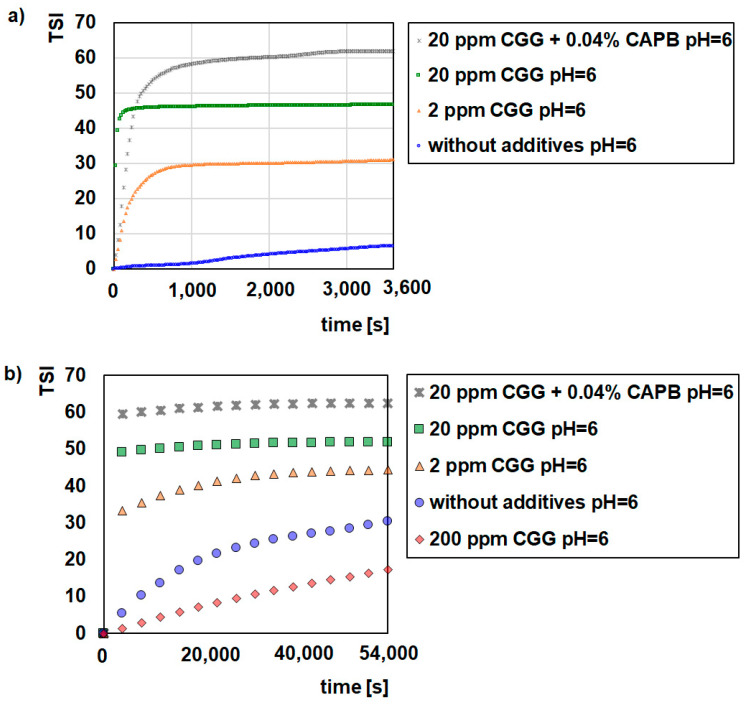
Influence of CGG with the addition of 0.04% CAPB on stability of the glauconite suspensions at pH = 6; 1 h (**a**), 15 h (**b**).

**Figure 3:**
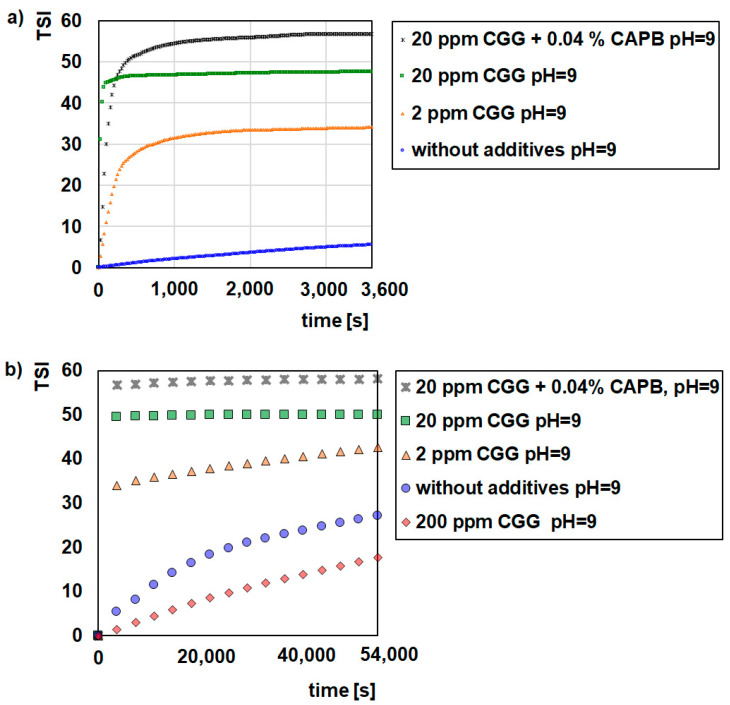
Influence of CGG with the addition of 0.04% CAPB on stability of the glauconite suspensions at pH = 9; 1 h (**a**), 15 h (**b**).

Figure 1, Figure 2 and Figure 3 illustrate the destabilization kinetics of the GT suspensions in the presence of CGG or a mixture of CGG and CAPB at different pH values (3, 6, or 9). For a better analysis of the suspension behaviour, the experiments were conducted for 15 h (scan every hour) as well as for 1 h (scan every 25 s) in systems where flocculation occurs. The obtained results show that pure glauconite suspended in a background electrolyte solution is very unstable at pH = 3, while at pH = 6 and pH = 9, it is quite stable. The evidence for that is small TSI values (Figure 1, Figure 2 and Figure 3) and large absorbance values as well as the small changes that these values undergo throughout the measurement process (Figure S1).

**Figure 4:**
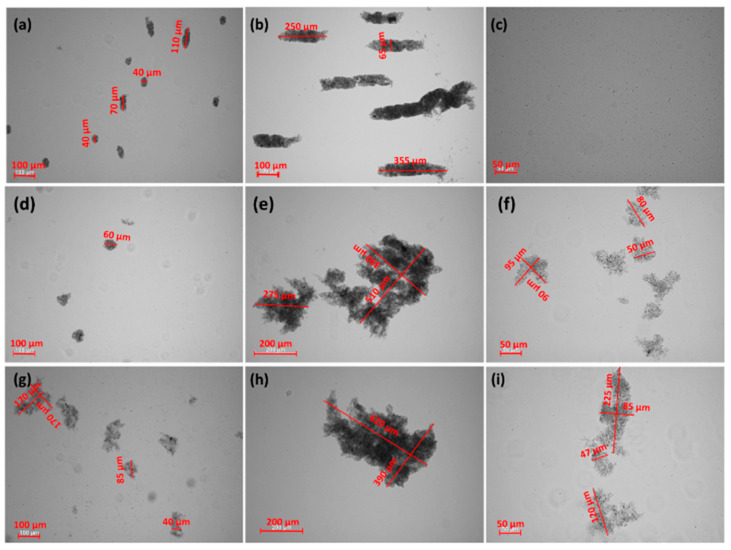
Optical microscope images of the GT flocs formed with varying CGG concentrations and pH values. IS 0.01 (NaCl): (**a**) 2 ppm of CGG, pH = 3; (**b**) 20 ppm of CGG, pH = 3; (**c**) 20 ppm of CGG, 0.04% CAPB, pH = 3; (**d**) 2 ppm of CGG, pH = 6; (**e**) 20 ppm of CGG, pH = 6; (**f**) 20 ppm of CGG, 0.04% CAPB, pH = 6; (**g**) 2 ppm of CGG, pH = 9; (**h**) 20 ppm of CGG, pH = 9; (**i**) 20 ppm of CGG, 0.04% CAPB, pH = 9.

The addition of CGG evidently changes the stability of the glauconite suspension. As the concentration of the polymer increases up to 20 ppm, the stability of the system deteriorates. Under this condition, after 60 min, the TSI values reached 49.07 and 49.49 at pH = 6 and 9, respectively, and hardly changed within 15 hrs. In comparison, the TSI for the polymer free system was approximately 5.5 at pH = 6 and 9. In the case of the suspension at pH = 3, the ∆TSI (14.7) between the control sample and the sample with 20 ppm of CGG was significantly lower compared to the sample at pH = 6 and 9. Moreover, with a smaller amount of biopolymer (2 ppm), the GT suspension was destabilized effectively at pH = 3. Regarding the size and morphology of the formed structures, it was determined that with the increasing CGG concentration, the flocs became bigger and denser (Figure 4). Additionally, at a low pH, the flocs had a completely different morphology, which was more elongated and packed. However, under such conditions the flocs were smaller than they were at a higher pH, which significantly affected the destabilization rate. The all of the GT particles were removed from the suspension within 250 (pH = 3), 100 (pH = 6), and 75 (pH = 9) seconds with the optimal polymer dosage, which was 20 ppm (Figure 1, Figure 2 and Figure 3). The larger the flocs, the faster they were able to sediment, and after 60 min, GT had a removal efficiency of more than 90% (Table 1). The changes in the sediment layer thickness (Table 2) are also in line with these findings. More sediment on the bottom sample indicates that more particles settled down when the biopolymer concentration increased in the suspension. This indicates strong flocculating properties of the CGG, whose efficiency largely depends on the system pH. An explanation for this phenomenon is the pH-dependent adsorption process of CGG, which takes place on the glauconite surface and causes flocculation [29,30].

Based on these results, it can be concluded that the mechanism that is most likely responsible for the flocculation process of aqueous glauconite suspensions is bridge flocculation [31,32]. This type of flocculation results from colloidal particles binding with the help of the polymer chains adsorbing on their surface [33,34]. Single polymer chain bridges extending between the surfaces of two or more colloidal particles hold the flocs. The polymers that are most preferred for bridge flocculation are the polymers with long “tails” hanging from the dispersed phase, and in addition, the amount that they are able to adsorb is below the saturation coverage value. In such a case when two particles collide, the free tail on the surface of one sticks to the free surface of the other. Several other processes also take place during bridge flocculation. Polymer chains are also able to mix between the colloidal particles, which results in polymer adsorption on these particles, various types of rearrangement of the adsorbed polymer as well as the formation and disintegration of the flocs [35,36]. It is because of this that GT can be easily separated from the system along with the adsorbed polymer molecules without additional steps such as filtration. However, the addition of a large amount of polymer (200 ppm) stabilized the suspension, as indicated by the small TSI values (Figure 1, Figure 2 and Figure 3) and high absorbance values (Figure S1). This situation results in a low degree of particle removal from the system (Table 1). 

The further addition of CAPB to a GT suspension containing 20 ppm of polymer had a different effect on the behaviour of the system that was dependent on the pH. At pH = 6 and 9, the suspensions were still unstable. However, the surfactant changed both the structure of the flocs and the rate of kinetic destabilization. The flocs were smaller and less packed, with more internal capillaries formed by the agglomeration of colloidal particles compared to those obtained in the system without the zwitterionic surfactant (Figure 4). This is why a longer time (approximately 1000 s) is needed for the complete removal of the GT particles from the investigated system. Additionally, the flocs that were created in the presence of CAPB formed loosely packed sediment, as evidenced by the higher sediment layer thickness compared to the samples without surfactant (Table 2). Interestingly, at pH = 3 in the presence of the surfactant the system also became stable with the addition of the polymer (20 ppm) (Figure 1). There were no visible GT agglomerates (Figure 4). It was found that this is due to the fact that stabilization only occurs in this system (GT/CGG/CAPB/pH = 3). In such a situation, the created polymer–surfactant complexes do not form aggregates of big flocs but are adsorbed on the GT surface, which causes the steric stabilization of the system [37].

### 2.2. Formation of the CGG/CAPB Complexes

**Figure 5:**
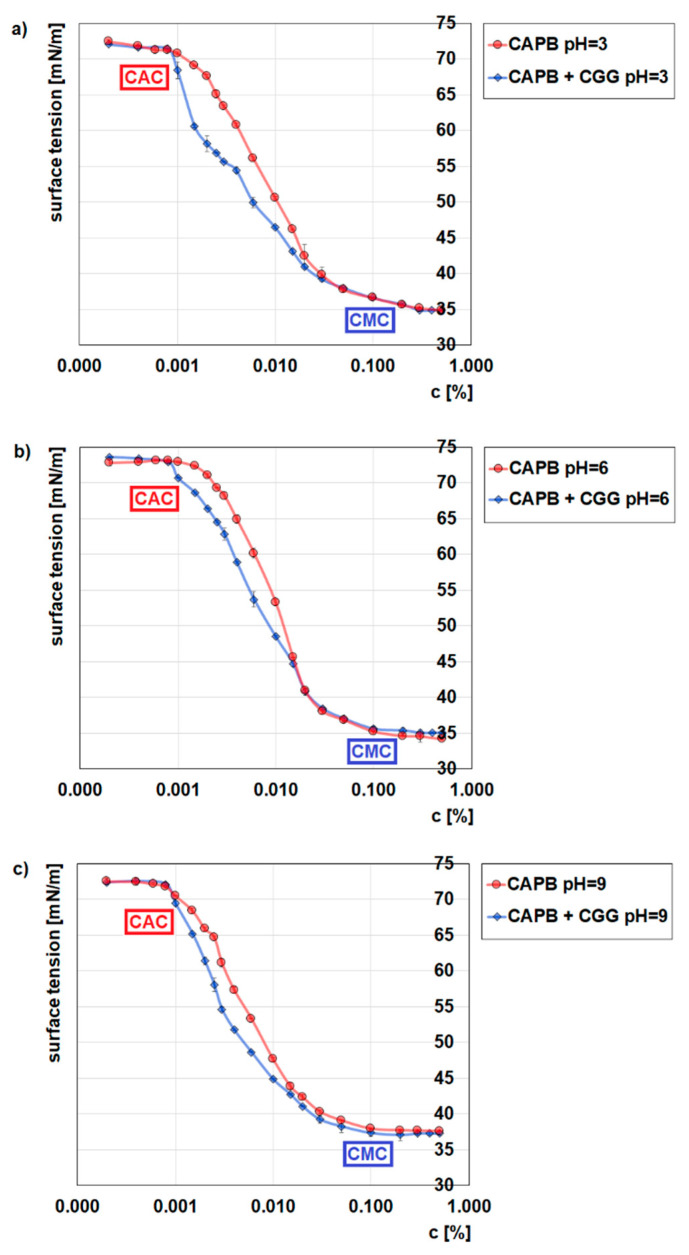
Influence of CGG (200 ppm) on the surface tension of CAPB at pH = 3 (**a**), pH = 6 (**b**), and pH = 9 (**c**).

Surface tension measurements and zeta potential measurements were used to confirm the formation of the polymer–surfactant complexes. Figure 5 shows the effect of the presence of CGG (200 ppm) on the surface tension of CAPB at various pH values. These measurements provide some information about the critical micellization concentration (CMC), the critical association concentration (CAC) and the saturation concentration (T_2_’). 

According to the obtained results, the value of the CMC for CAPB does not depend on pH and equals about 0.1%, which is consistent with the literature data [38]. In the systems with CGG where strong interactions between CAPB and CGG are observed, the values of CMC and T_2_’ cannot be determined [39,40]. The results show also that the formation of the intermolecular polymer–surfactant complexes between CGG and CAPB takes place. Evidence for this includes the differences that can be observed to take place over the course of the CAPB surface tension curves in the presence and absence of CGG.

Figure S2 presents the influence of the CAPB concentration on the zeta potential of CGG at various pH values. As one can see, the formed complexes are positively charged at every studied pH. Their charges are the lowest at pH = 3, medium at pH = 6, and the highest at pH = 9, which were caused by the cationic character of CGG and the influence of the pH on the dissociation degree. The characteristic increases in the zeta potential of the CGG/CAPB complexes at every measured pH value correspond to the critical association concentrations (CAC), where the effective formation of CAPB/CGG complexes starts.

### 2.3. Adsorption and Electrokinetic Measurements

**Figure 6:**
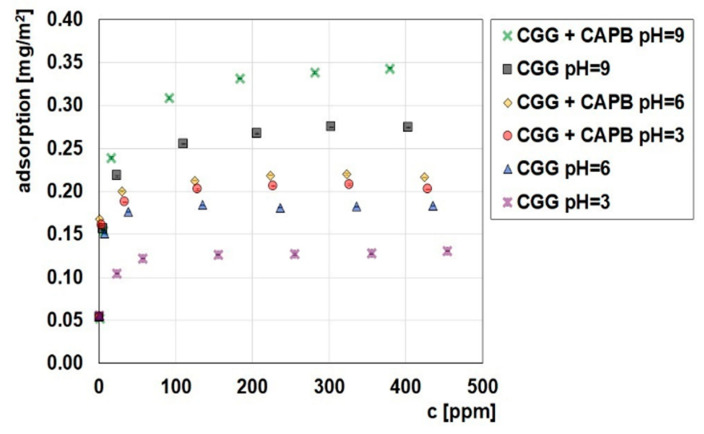
Influence of CAPG (0.04%) on the CGG adsorption on the glauconite surface at different pH values.

**Figure 7:**
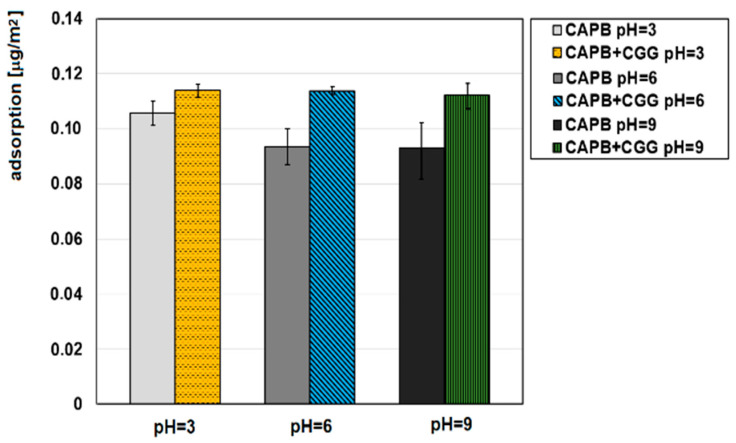
Influence of CGG (200 ppm) on the CAPB adsorption (0.04%) on the glauconite surface at different pH values.

In order to analyse the flocculation mechanism, studies determining the adsorption capabilities of CCG on the glauconite surface were conducted. Figure 6 shows the CCG adsorption isotherms either without or with the addition of CAPB (0.04%) on the GT surface at various pH values, while Figure 7 provides the adsorption isotherms of CAPB with the addition of CGG (200 ppm) on the GT surface at various pH values. It can be seen from both figures that the pH has a very large influence on the amount of adsorption. It can be concluded from Figure 6 that the CCG adsorption amount increases with the increase of pH. The cationic guar gum adsorbs fairly well to the negatively charged surface of the glauconite, and the adsorption plateau is quickly established. The characteristic of the adsorbent surface was confirmed by the study of the zeta potential of the polymer/glauconite suspension (Figure 7). The obtained results show that glauconite is negatively charged in the entire pH range, while when the polymer concentration increases, the charge is neutralized as the effect of the CGG electrostatic adsorption. When the pH of the system increases, the electrostatic attraction between the negatively charged adsorbent and the positively charged polymer also increases, which causes the CCG adsorption on CT to increase [41]. After the addition of the zwitterionic CAPB to the adsorption system, the situation changes. Its presence causes a significant increase in the amount of the CGG that can be adsorbed. The reason for this is the formation of multimolecular polymer–surfactant complexes. The complexation process was confirmed by the surface tension measurements (Figure 5). The adsorption of the formed complexes consisting of a few macromolecules of CGG joined with the surfactant molecules causes the total amount of adsorption to be higher compared to in the systems without the surfactant. 

The results of the amount of CCG adsorption after the addition of CAPB (0.04%) on the GT surface were confirmed by the BET analysis. The obtained data (Table 3) show that in the case of a pure adsorbent, the specific surface area and pore volume have the highest values. On the other hand, the greater the adsorption amount of the polymer or the polymer/surfactant complexes, the smaller the values of the specific surface area and the pore volume of glauconite are. The results in Table 3 clearly show that the mesopores on the GT surface are partly blocked due to adsorption of those organic species.

Figure 7 shows that the CAPB adsorption occurs in both the presence and absence of CGG on the glauconite surface. It is known that betaines are uncharged at neutral pH and exhibit a positive charge under the small pH value. However, under a large pH value, the presence of the net positive charge around the nitrogen atom causes the CAPB molecules to never become anionic. It can be seen that CAPB adsorbs on GT readily at every studied pH value and that its adsorption is the greatest when the pH equals 3. The reason for this is that the largest possible positive charge causing the effective electrostatic adsorption on the negatively charged GT. The CAPB adsorption amount on GT at pH 6 and 9 is similar, which proves that CAPB is a pseudoamphoteric surfactant. In this case, CAPB was a truly amphoteric substance, so there should have been a decrease in the adsorption on the negatively charged glauconite at a high pH. Another observation from the obtained data is that the addition of CGG to the adsorption system causes CAPB adsorption to increase. This fact results from the above-mentioned formation of the multimolecular polymer–surfactant complexes and their adsorption on the glauconite surface. This finding is consistent with the previously presented adsorption data. The comparison of the adsorption amount of CGG in the presence of CAPB (Figure 6) and the adsorption amount of CAPB in the presence of CGG at different pH values (Figure 7) shows that the formed complexes are adsorbed the most effectively at pH = 9, while their adsorption at smaller pH values is comparable. It is also possible that the complexes that are formed at pH = 9 have a larger number of the polymer macromolecules compared to those prepared at smaller pH values; however, the number of surfactant molecules included in the complexes remains similar regardless of pH. 

**Figure 8:**
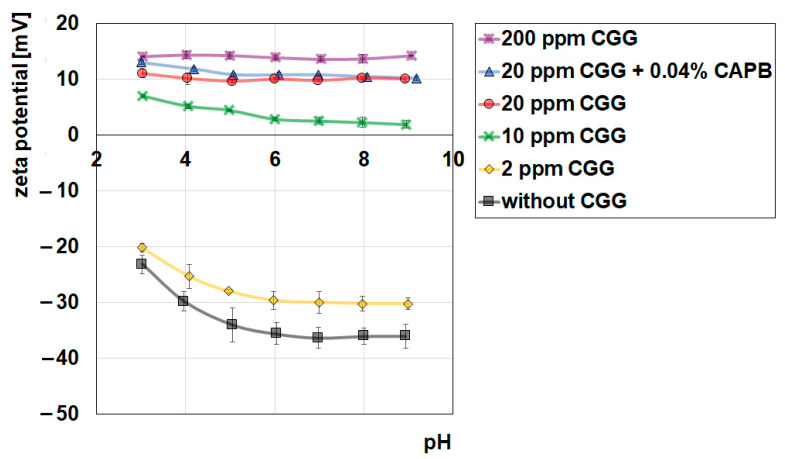
Influence of CGG and CAPB (0.04%) on the zeta potential of glauconite.

Figure 8 shows the effect of CGG and CAPB (0.04%) on the zeta potential of glauconite. The zeta potential (ζ) is the electrokinetic potential that is measured at the slipping plane. The zeta of pure glauconite is negative in the whole measured pH range, which is typical of mineral clays [42,43], and decreases when the pH increases. However, the addition of CGG causes the increase of the zeta potential. Such behaviour usually occurs in systems with cationic macromolecules [44] where the factor connected with the charge and that influences the zeta potential surpasses the other factors (for example the shift of the slipping plane). This increase of the zeta potential is larger when the CGG concentration increases. This can be explained by the presence of the positively charged groups from the CGG macromolecules. When the zeta potential is measured in the CGG/CAPB/GT system, a slight increase in the zeta potential is observed compared to the system without the surfactant. This is the effect of additional charge in the diffused part of the electrical double layer from the CAPB molecules.

**Figure 9:**
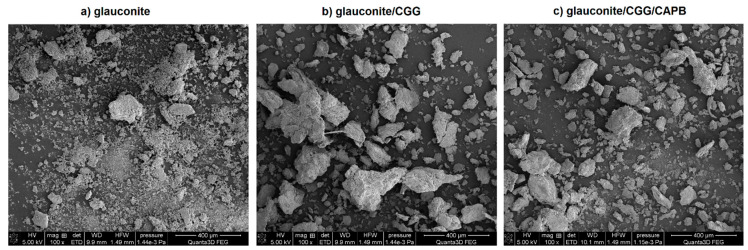
SEM micrographs differences in the morphology of glauconite before and after the adsorption of CGG and CGG with CAPB: (**a**) pure glauconite, (**b**) glauconite/CGG, (**c**) glauconite/CGG/CAPB; magnification ×100.

In order to analyse the changes in the GT morphology before and after the adsorption of CGG or CGG and CAPB complexes as well as for the quantitative analysis of the surface composition of these systems, the SEM/EDS technique was used. Figure 9 presents the SEM micrographs showing the changes in the morphology of the studied systems at pH = 6. The SEM image (Figure 9a) shows the GT sample to be a mix of several big grains surrounded by a large amount of fine debris. As one can see, the adsorption of CGG on GT changes the granulation of the adsorbent completely. The sizes of the particles are visibly larger, which is the effect of the flocculation occurring in the system. As far as the glauconite/CGG/CAPB system is concerned, it is clear that the mean particle size is smaller compared to the previously mentioned system. These findings correspond well to the TSI data as well as to the optical microscope analysis presented in Figure 2 and Figure 4, respectively.

**Figure 10:**
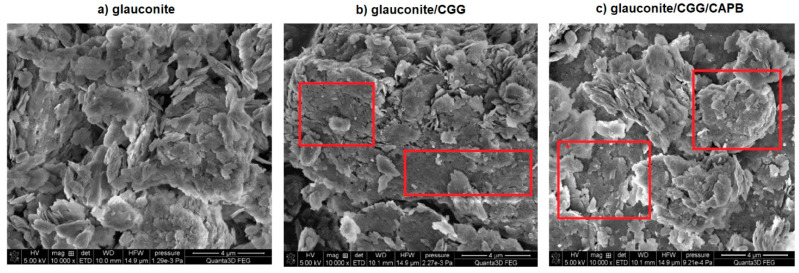
SEM image of glauconite: (**a**) pure glauconite, (**b**) glauconite/CGG, (**c**) glauconite/CGG/CAPB; magnification: ×10,000.

As it can be seen on Figure 10b,c, the lamellar structure of the grains after the organic treatment is more compacted, and gaps along the crystal boundaries are covered with the biopolymers or the CGG/CAPB complex (see the areas in the red boxes), resulting in these materials having a smaller porosity.

The quantitative changes in the surface composition of glauconite during the adsorption of the CGG and CGG/CAPB complexes are presented in Table 4. The chemical formula for pure glauconite is (K,Na,Ca)(Fe,Al,Mg)_2_[(OH)_2_/(Al,Si)_4_O_10_] × nH_2_O, as previously mentioned. The obtained results are generally consistent with the theoretical composition of this mineral. The additional presence of carbon is probably a result of the adsorption of carbon dioxide on the glauconite surface. Moreover, the studied glauconite sample also contained small amounts of Ti, which is quite a common impurity in glauconite samples [45,46]. In the GT/CGG system, the nitrogen and chlorine atoms from the CGG macromolecules appear, and the amount of carbon and oxygen increases, whereas in the GT/CGG/CAPB system, the amount of carbon, nitrogen, and oxygen is the largest, which is an expected effect of the CAPB presence. In addition, a significant decrease in the content of Si and metals in the modified samples confirms the adsorption of CGG and CAPB on the glauconite surface.

### 2.4. FT-IR Measurements

**Figure 11:**
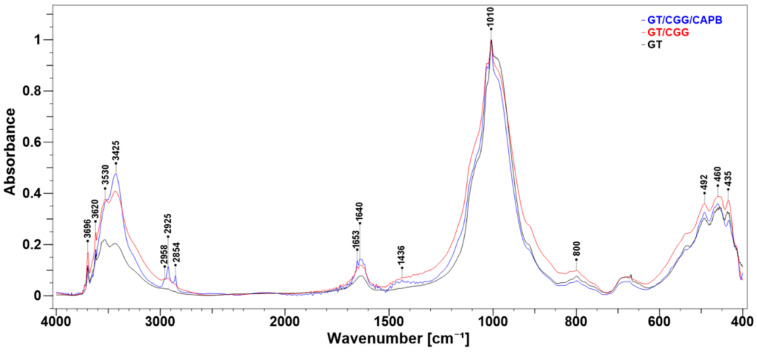
FTIR spectra of natural clay (GT) and composites: GT/CGG and GT/CGG/CAPB. All spectra were normalized on the band at 1010 cm^−1^.

Figure 11 depicts the FTIR spectra of the natural GT clay and the organically modified samples (organoclays). Their comparison shows that the organoclays retain the profile of the starting clay spectrum whose major bands occur in the OH- stretching vibration region (3700–3400 cm^−1^) and in the Si–O/Al–O stretching (1100–900 cm^−1^) and Si–O/Al–O bending vibration areas (600–400 cm^−1^) [45,47]. This observation indicates that the crystalline structure of GT remained unchanged after the modifications made to CGG and CAPB. However, an obvious increase in the relative intensities of the absorption in the OH stretching region of organoclays between 3696 cm^−1^–3425 cm^−1^ can be observed. The weak and sharp bands at 3696 cm^−1^ and 3420 cm^−1^ were assigned to the stretching vibrations of the surface and inner hydroxyl groups in kaolinite, respectively [48,49,50,51]. The former of two poorly resolved bands at 3530 cm^−1^ was assigned to the structural OH−stretching mode in largely disordered glauconite, while the later one at 3425 cm^−1^ was attributed to the OH−stretching of the interlayer and adsorbed water [49,50]. An absorption corresponding to the bending vibrations of the H_2_O molecules occurs at 1620 cm^−1^ as a single band. This increase could be due to the overlapping of the O−H and N−H amide stretching of the CGG and CAPB functional groups. Moreover, the FTIR spectra of the composites reveal the new absorption bands at 2959, 2925, 2854, 1652, 1640, and ~1436 cm^−1^. The bands at 2959, 2925, and 2854 cm^−1^ can be attributed to the asymmetric stretching vibrations of C−H in the alkyl chain, while the band at 2854 cm^−1^ and the hump centred at 1436 cm^−1^ can be attributed to the symmetric stretching and bending modes, respectively, of the same alkyl group as well as to the scissoring of N^+^−CH_3_ moiety [52,53,54,55]. The unresolved peaks around 1640 cm^−1^ correspond to the stretching vibration of the associated C=O groups (amide I band of secondary amides) and COO^−^ groups in the zwitterionic surfactant [56,57]. The spectral data confirm an aggregation of the CGG biopolymer and the CAPB surfactant on the GT surface. 

### 2.5. Powder X-ray Diffraction Measurements 

**Figure 12:**
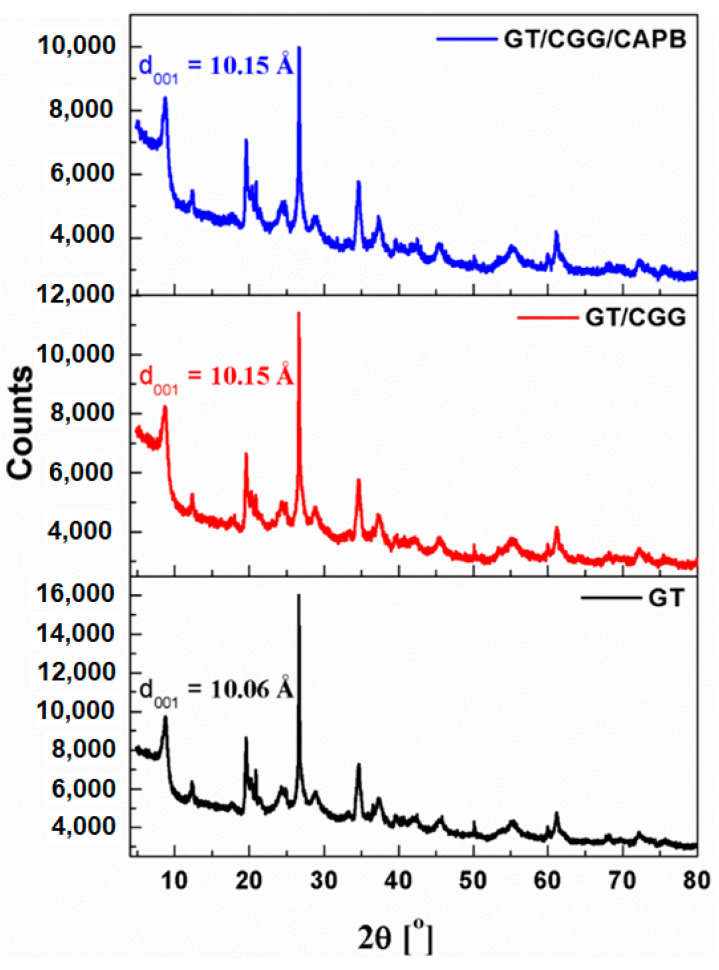
X-ray diffraction patters of glauconite clay GT and GT modified by CGG and the CGG/CAPB mixture.

XRD random powder analysis was chosen to evaluate the nature of the interactions between the CGG and CGG/CAPB species and glauconite GT. All of the diffractograms (Figure 12) display the 10 Å diffraction peak, which is characteristic for the d_001_ basal spacing (2:1 layer + interlayer) of the mica-like minerals [58,59,60,61].

Glauconite, similar to kaolinite, belongs to the group of non-expanding minerals. The isomorphic substitution of the silicon sites with aluminium atoms (about 20%) is the main source of a negative net charge in the glauconite tetrahedral sheet [60]. Potassium ions that are sufficiently sized to fit into the spaces of the adjacent tetrahedral sheets are strongly attracted to the interlayer to balance this charge. In this way, they act as a natural binder, preventing water and organic ions from infiltrating between packets and crystal expansion. In turn, kaolinite has little or even no electric charge layers, and therefore, no large cations (such as calcium, sodium, or potassium) are present between the structural layers. Moreover, the tight hydrogen bonding between the gap sheets fixes the structure, and no expansion commonly occurs between the layers when the clay is wetted. Consequently, the effective surface of glauconite and kaolinite should be restricted to its edge or to its external surface area [62,63,64,65]. The structure of glauconite usually contains variable amounts of expandable clay interlayers [66,67,68]. However, the curves in Figure 12 show that the layered structure of clay was not affected by the treatment, and no detectable changes on the d_001_ interlayer spacing of the organically modified samples were observed. This suggests that the layered structure of GT contains no or only few expandable (smectite) layers. Thus, according to the XRD patterns and the FTIR study, the organic species (CGG and CAPB) are not intercalated into the interlayer of the GT clay but are rather adsorbed on its surface. The length of the alkyl chain of CAPB and a highly branched structure of CGG polysaccharide could also hinder the intercalation of the glauconite interlayers. On the other hand, the surface charge of glauconite and kaolinite (if any) could promote the adsorption of CGG and CAPB species on the surface via the electrostatic interactions. Therefore, the adsorption of zwitterionic CAPB and cationic CGG on the GT surface most probably occurs via two consecutive mechanisms: electrostatic interactions and hydrophobic interactions. A similar mechanism was reported upon the modification of vermiculite by the amphoteric surfactants [49,69] and kaolinite modified with different alkylammonium salts [70,71].

## 3. Materials and Methods

### 3.1. Materials 

Jaguar^®^ Excel cationic guar gum (CGG, guar hydroxypropyltrimonium chloride, N-(3-chloro-2-hydroxypropyl) trimethylammonium chloride), CAS = 65497-29-2, which was used as an adsorbate, was purchased from Solvay. The chemical structure of this compound is presented in Figure S3. The results of the elemental analysis of this polysaccharide (EuroEA3000 CHNS-O Analyser, EuroVecor, Pavia, Italy) are as follows: N% = 1.599, C% = 37.916, H% = 6.480. It was found that there is a considerable percentage of nitrogen in CGG that can be accounted for by the presence of CHPTAC ((3-Chloro-2-hydroxypropyl)trimethylammonium chloride) chains on the polysaccharide backbone. The molecular weight of CGG was estimated using the Zimm plot and was found to be 2.17 × 105 g/mol (Zetasizer NanoZS, Malvern Instruments, Worcestershire, UK). The CGG stock solutions were prepared by the fast addition of CGG into vigorously stirred re-distilled water and further stirring for 30 min. In order to avoid degradation, the obtained solution was used within a one week period.

TEGO^®^ Betain CK D (CAPB) of the formula: C_19_H_38_N_2_O_3_ was purchased from Evonik Operations GmbH. The results of elemental analysis of the polysaccharide (EuroEA3000 CHNS-O Analyser, EuroVecor, Pavia, Italy) are as follows: N% = 6.660, C% = 56.695, H% = 9.504. The chemical structure of this compound is presented in Figure S3.

Glauconite, which was extracted from natural sources in the Lublin Upland, was used as the adsorbent. The specific surface area of 70.21 m^2^/g, the pore volume of 0.1644 cm^3^/g, and the mean pore diameter of 9.37 nm were determined by means of the BET method (ASAP 2405 analyzer, Micrometritics, GA, USA). The particle size distribution (d0.1 = 1.180 µm; d0.5 = 7.755 µm; d0.9 = 36.727 µm) was also determined (DLS method, Mastersizer 2000, Malvern Instruments, Worcestershire, UK). The results of the elemental analysis of the polysaccharide (EuroEA3000 CHNS-O Analyser, EuroVecor, Pavia, Italy) are as follows: N% = 0.071, C% = 0.228, H% = 0.712. The XRD pattern and phase analysis by the Rietveld method shows that the neat clay mainly comprises glauconite (81%) with quartz (15%) and a small admixture of kaolinite (5%) (Figure S4, Table S1).

**Figure 13:**
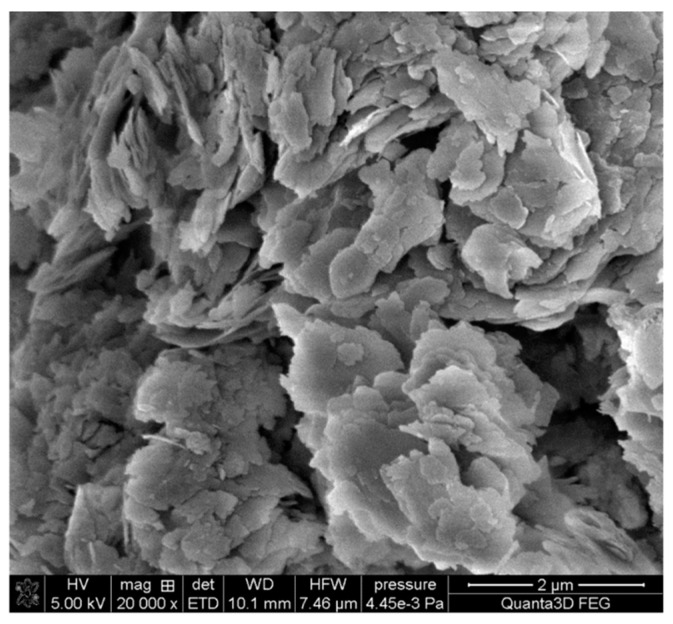
The SEM micrograph showing the morphology of the used glauconite, magnification ×10,000.

In order to ensure the largest specific surface area and to remove any contamination, prior to testing, glauconite was washed with re-distilled water until the conductivity of the filtrate was smaller than that of pure re-distilled water (about 2 μS/cm). Moreover, the scanning electron microscopy (SEM) was used to analyse its morphology (Figure 13). The results were obtained by SEM Quanta 3D FEG (FEI, CO). The voltage was 5 KV, and the micrograph was taken at a magnification of ×10,000. A SEM image shows that the aggregates of glauconite grains mostly consist of loosely arranged flaky particles (platelets) ranging in mean size from 0.25 to 1 μm. The platelets of glauconite layers pile up, forming tectoid-like particles in which the wedge-like splitting of glauconitic layers creates the open and mesopore structure (Figure 13).

### 3.2. Methods

#### 3.2.1. Stability Measurement

The stability of the studied systems was monitored using the turbidimetric method (Turbiscan LAB Expert, Formulation). These measurements are based on monitoring the laser light intensity before and after it passes through the studied system (λ = 880 nm). The device is equipped with two detectors and registers the transmittance (0°) and the backscattering (135°) of the laser light. The great advantage of this method is the fact that the laser head scans the entire test-tube from the top to the bottom. This allows the calculation of the turbiscan stability index (TSI) and the sediment layer thickness. The TSI changes from 0 to 100, where the higher the value, the more unstable the sample is. The sediment layer thickness, as a peak thickness of the sediment layer formed at the bottom of the vial, was computed from the backscattering (BS) profiles of the samples. The samples were prepared with the addition of 0.003 g of GT to the prepared solution (background electrolyte NaCl and re-distilled water). The pH of the suspensions was set to 3, 6, or 9 using either HCl or NaOH. Then, the prepared suspensions were treated by ultrasound for 20 s (Sonics Vibra Cell ultrasonicator, Sonics & Materials, Inc., Newtown, CT, USA, 20 s), and the polymer (CGG: 2, 20 or 200 ppm) or the polymer (CGG: 20 ppm) and surfactant (CAPB: 0.04% (*w*/*w*)) were added. After mixing, the sample was placed in the device. To observe flocculation and the systems where the flocculation process occurs, the changes in the system stability were monitored every 25 s for 1 h and every 1 h for 15 h. In addition, the floc morphology and the size were evaluated by optical microscope analysis (AxioImager.M1m, Zeiss, Oberkochen, Germany). To determine the nanoparticle removal efficiency, the optical densities (OD_500_) of the supernatant at 500 nm were measured using a spectrophotometer (UV-Vis Evolution 201, Thermo Fisher Scientific, MA, USA). Based on the calibration curve (OD_500_ versus nanoparticles concentration), the concentration of the nanoparticles in the supernatant was determined. 

The removal efficiency was calculated form Formula (1).

(1) $$R=\frac{\left(C^{o}-C\right)}{C^{o}}\times 100$$

where *C^o^* is an initial concentration of the GT (ppm), *C* is a concentration of the GT in the supernatant after 1 h.

In order to confirm the obtained results, comparative measurements were performed using the spectrophotometric method. The prepared solutions contained 0.003 g of glauconite, 1 cm^3^ of the background electrolyte (NaCl) at a concentration of 0.01 mol/dm^3^, and the appropriate volume of redistilled water so that the total volume was 10 cm^3^. The solutions that were prepared in this way were sonicated (Sonics Vibra Cell ultrasonicator, Sonics & Materials, Inc., Newtown, CT, USA, 20 s). The next step was the addition of the appropriate volume of CGG so that its concentration was 2, 20, or 200 ppm. An appropriate volume of CAPB was added to the solution with a concentration of 20 ppm CGG so that its concentration was 0.04% (*w*/*w*). The last step was to adjust the pH value to 3, 6, or 9. The suspension that was prepared in this way was placed in a quartz cuvette, which was put into in the spectrophotometer, and the sample was scanned for 15 h.

#### 3.2.2. Formation of the CGG/CAPB Complexes

The surface tension was determined based on the tensiometric measurements using the pedant drop method. Measurements were made using a Theta optical tensiometer (KSV Instruments Ltd., Helsinki, Finland). Twenty flasks with a volume of 50 cm^3^ each were prepared for this purpose. The appropriate volume of CAPB was placed into each flask to obtain surfactant concentrations in the range of 0.001–0.5% (*w*/*w*) in each flask. For the series of the measurements preformed in the presence of CGG, the appropriate amount of this polymer was added to each flask so that its concentration equalled 200 ppm, and the concentrations of CAPB were in the same range as they were previously. Each measurement was repeated five times, and the average values were reported.

The zeta potential of CGG with and without CAPB were measured using a Zetasizer Nano ZS (Malvern Instruments, Worcestershire, UK). Samples containing CGG (200 ppm) or CGG (200 ppm) and surfactant (the concentration ranges: 0.0002–0.5% (*w*/*w*)) were prepared, and the pH was adjusted to 3, 6, or 9. The electrophoretic mobility was measured and was recalculated to the zeta potential using the Smoluchowski equation.

#### 3.2.3. Adsorption Measurements

To determine the amount of CGG adsorption, the colorimetric method described by Dubois et al. [72] was used. The measurements started with the determination of the calibration curves. For this purpose, solutions of CGG with concentrations of 20, 60, 100, 200, 300, 400, and 500 ppm containing the background electrolyte NaCl of 0.01 mol/dm^3^ were prepared in 10 cm^3^ flasks. The pH was adjusted to 3, 6, or 9. From each flask, 1 cm^3^ of the solution was taken and placed in a test tube, to which 1 cm^3^ of re-distilled water, 0.05 cm^3^ of 80% (*w*/*w*) phenol, and 5 cm^3^ of concentrated sulfuric acid (VI) were added. After 30 min. of colour development, the spectrophotometric measurements were made at a wavelength of 490 nm. Each measurement was repeated four times. 

After the determination of the calibration curves, the CGG adsorption was measured in the absence and presence of 0.04% (*w*/*w*) CAPB on the glauconite surface at various pH values (3, 6, or 9). For this purpose, the solutions were prepared in an analogous manner to those of the calibration curves in conical flasks with a volume of 25 cm^3^. However, glauconite of 0.05 g was added to each of them, and the obtained suspensions were shaken for 24 h. After this time, the suspensions were centrifuged twice for 15 min using a laboratory centrifuge. The amount of adsorption was determined by the spectrophotometric method at a wavelength of 490 nm. Each measurement was repeated four times, and the average values were reported.

The measurements of the amount of CAPB adsorption on the glauconite surface at different pH values also started with the preparation of the calibration curves. For this purpose, eight solutions were prepared in 10 cm^3^ flasks containing surfactant (0.02, 0.04, 0.1, 0.5, 0.7, 1, 3, and 5% (*w*/*w*)) and re-distilled water. One set of measurements was made in the presence of CGG (200 ppm). From the obtained solutions, 5 cm^3^ of the sample was moved into Erlenmayer flasks, 5 cm^3^ of the acetate buffer, 10 cm^3^ of redistilled water, and 5 cm^3^ of methanil yellow at a concentration of 10^−3^ mol/dm^3^ were added. Then, 10 cm^3^ of chloroform was added to each flask, and the solutions were shaken for 5 min and were then left to stand for 20 min. After this time, the chloroform layer was collected with a pipette, placed in centrifuge tubes, and centrifuged for 2 min using a laboratory device. Then, the spectrophotometric measurements were made at a wavelength of 404 nm.

The CAPB adsorption amount on the surface of glauconite in the absence and presence of CGG (200 ppm) was measured. To this end, the solutions were prepared in eight Erlenmayer flasks as they were for the calibration curves, but 0.05 g of glauconite was added to each of them, the pH was adjusted to 3, 6, or 9, and the obtained suspensions were shaken for 24 h. After this time, the suspensions were centrifuged twice for 15 min. The amount of CAPB adsorption was determined spectrophotometrically at a wavelength of 404 nm. Each measurement was repeated four times, and the average values were reported.

Scanning electron microscopy (SEM) was used as a supporting technique to analyse the changes in the morphology of the GT at pH = 6 before and after the adsorption of the CGG or CGG/CAPB complexes as well as for the quantitative analysis of the surface composition (the average from three measurements). The results were obtained by SEM Quanta 3D FEG (FEI, CO). The voltage was 5 KV, and the micrograph was taken at a magnification of ×2000

#### 3.2.4. Electrokinetic Measurements

The solutions were prepared in a beaker with a volume of 500 cm^3^, in which 50 cm^3^ of the background electrolyte of 10^−2^ mol/dm^3^ NaCl and 0.05 g of previously weighed glauconite were placed. The suspension was sonicated (Sonics Vibra Cell ultrasonicator, Sonics & Materials, Inc., Newtown, CT, USA, 3 min). After ultrasonication, the system was split into seven small portions, and the pH was adjusted to 3–9. Before each measurement, the cells were rinsed with the studied solution. The device was a NanoZS Zetasizer (Malvern Instruments, Worcestershire, UK). The software converted the electrophoretic mobility of the tested sample into the zeta potential using the Smoluchowski equation. In the case of the solutions with CGG, the appropriate polymer was added to the suspension after ultrasonication to provide the concentrations of 2, 10, 20, and 200 ppm. As for the surfactant measurements, 0.04% (*w*/*w*) CAPB was added to the glauconite suspension with 20 ppm of CGG. Each measurement was repeated four times, and the average values were reported.

#### 3.2.5. FT-IR Spectroscopy

Infrared spectra using the KBr pressed disc method were acquired with a Nicolet 8700 FTIR spectrometer (Thermo Scientific, Waltham, MA, USA) in the scanning range of 4000–400 cm^−1^. For each spectrum, 64 scans were averaged. The normalization and the baseline correction functions were used.

#### 3.2.6. Powder X-ray Diffraction Study

The diffraction data were collected using an Empyrean diffractometer (PANalytical, The Netherlands) with a PIXcel3D detector using monochromated Cu-Kα radiation (λ = 1.542 Å) in a scan range from 5 to 80° 2θ with a 0.02° 2θ step increment.

## 4. Conclusions

Cationic guar gum can be used as the effective flocculant of glauconite. This finding is very important in terms of using such systems commercially, for example, for water purification. The most likely mechanism that is responsible for flocculation is bridge flocculation, which results from the colloidal particles sticking together by the polymer chains adsorbing on their surface. The adsorption process is caused by the strong electrostatic interactions between the anionically charged glauconite surface and the cationically charged polymer. The amount of CGG adsorption increases when the pH increases, which was confirmed by the studies of the zeta potential (when the CGG concentration increases, the zeta potential becomes more positive). The addition of the pseudoamphoteric surfactant CAPB increases the amount of the CGG adsorption on the GT surface due to the formation of the intermolecular polymer–surfactant complexes. The presence of such complexes was confirmed by the surface tension and zeta potential studies. The conducted measurements of the significant effect of pH and the presence of CAPB on the flocculation of the aqueous CGG/GT suspensions determine the mechanisms of the occurring processes in the studied systems and provide light for the optimization of these processes for the potential industrial applications. Apart from formation of the smaller flocs in the presence of CAPB, the destabilization rate of the GT/CGG system at pH above 6 is satisfactory. This finding proves that glauconite can be successfully used as a sorbent for removing polymers and their mixtures with surfactants in the cosmetic and pharmaceutical industries. In subsequent studies, the adsorption process of the CGG and the CGG/CAPB molecules on the GT surface will be further investigated in terms of optimization, kinetics, and thermodynamics.

Highlights:

- Glauconite (GT) can be used as water purifying material.
- CGG is an effective flocculant for glauconite suspensions.
- CAPB increased the CGG adsorption on the glauconite surface but deteriorated flocculation.
- Intermolecular polymer–surfactant complexes between CGG and CAPB were formed.
- pH strongly influences the stabilizing flocculating properties of the GT suspensions.

## Figures and Tables

**Table 1:** The influence of the pH and the CGG concentration on GT particle removal from the investigated system (IS 0.01) after 1 h.

CGG (ppm)	CAPB (%)	GT Removal (%)		
		pH = 3	pH = 6	pH = 9
0	0	76.2	34.7	36.0
2	0	96.5	68.4	71.9
20	0	99.9	97.8	99.6
20	0.04	9.0	98.5	99.2
200	0	10.9	10.6	10.2

**Table 2:** The influence of the pH and the CGG concentration on the sediment layer thickness in the studied systems after 1 h.

CGG (ppm)	CAPB (%)	Sediment Layer Thickness (mm)		
		pH = 3	pH = 6	pH = 9
0	0	1.01	0.44	0.45
2	0	1.19	0.77	0.65
20	0	1.28	1.01	1.01
20	0.04	-	1.26	1.34

**Table 3:** The BET analysis of pure and the modified glauconites.

	GT	GT + CGG	GT + CGG + CAPB
BET surface area (m^2^/g)	70.73	56.06	16.53
Total pore volume (cm^3^/g)	0.1691	0.1519	0.1015

**Table 4:** The qquantitative analysis of the surface composition of pure and modified GT by SEM.

	GT (wt. %)	GT/CGG (wt. %)	GT/CGG/CAPB (wt. %)
C	8.81 ± 0.39	13.27 ± 0.19	17.66 ± 0.79
O	44.1 ± 1.51	45.27 ± 0.29	45.60 ± 0.49
Mg	1.62 ± 0.05	1.45 ± 0.41	1.36 ± 0.24
Al	4.81 ± 0.27	4.37 ± 1.04	3.87 ± 0.69
Si	20.1 ± 0.90	16.66 ± 0.32	15.07 ± 0.87
K	5.38 ± 0.68	4.38 ± 0.70	3.37 ± 0.51
Ca	0.42 ± 0.03	0.42 ± 0.04	0.30 ± 0.05
Ti	0.2 ± 0.07	0.12 ± 0.09	0.07 ± 0.07
Fe	14.36 ± 1.72	11.47 ± 0.58	8.64 ± 0.40
Na	0.1 ± 0.04	0.08 ± 0.06	0.43 ± 0.07
N	-	1.99 ± 0.09	2.73 ± 0.22
Cl	-	0.95 ± 0.08	1.12 ± 0.60

## Data Availability

Data is contained within the article or Supplementary Materials.

## Supplementary Materials

The following are available online at https://www.mdpi.com/article/10.3390/ijms222212157/s1.
