# Supplementary material for: Influence of Zwitterionic CAPB on Flocculation of the Aqueous Cationic Guar Gum/Glauconite Suspensions at Various pH
Source: Int J Mol Sci. 2021 Nov 10;22(22):12157. doi: 10.3390/ijms222212157 (PMC8621159; doi:10.3390/ijms222212157)
Supplement: Supplementary file 1 [file ijms-22-12157-s001.zip › ijms-1421562-supplementary.pdf]

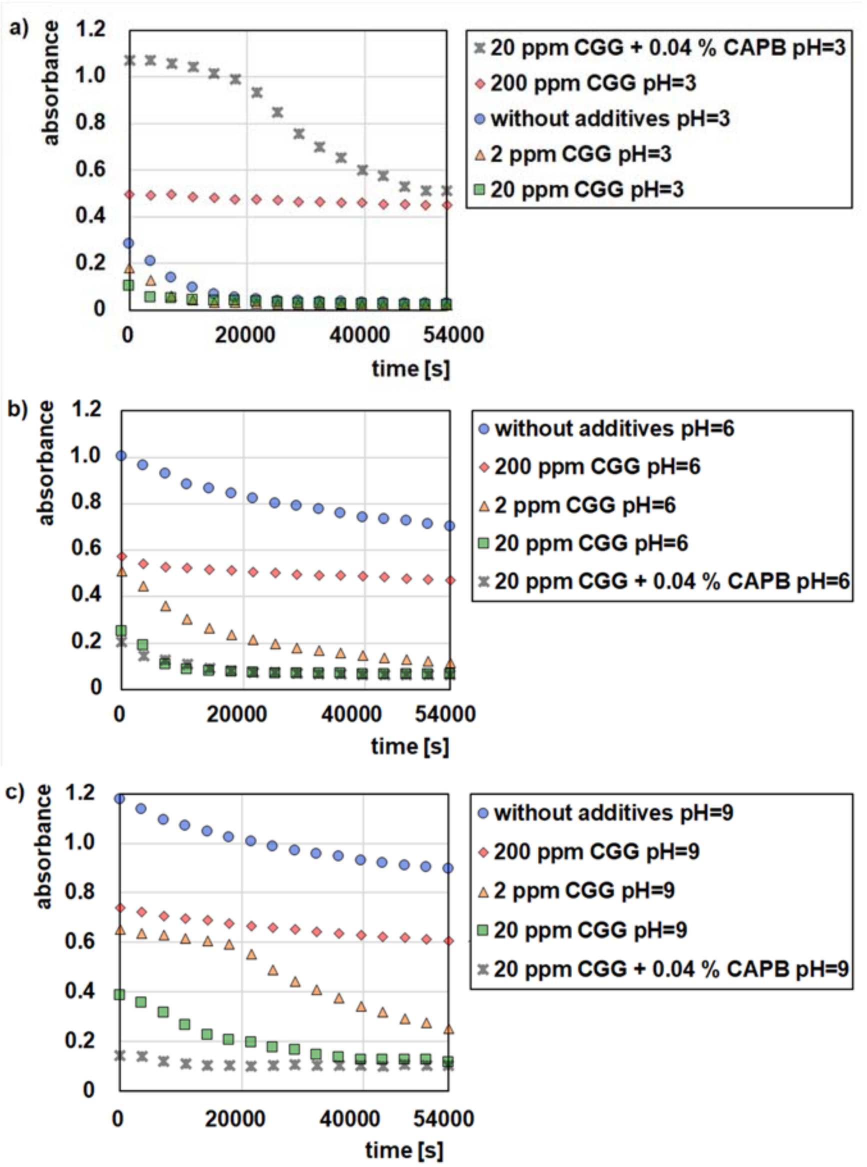

Figure S1. Influence of CGG on stability of the glauconite suspensions at pH=3 (a), pH=6 (b) and pH=9 (c).

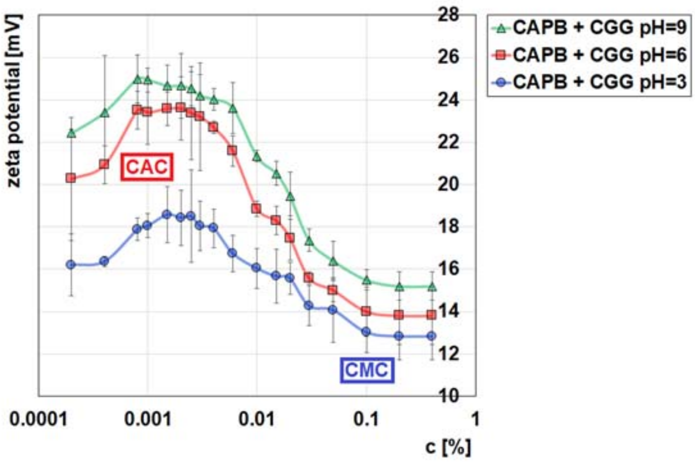

Figure S2. Influence of CAPB on the zeta potential of CGG (200 ppm) at pH=3, 6 and 9.

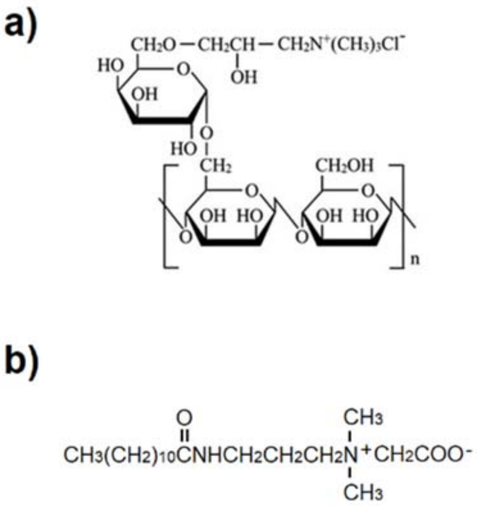

Figure S3. Chemical structures of the compounds: a) CGG, b) CAPB.

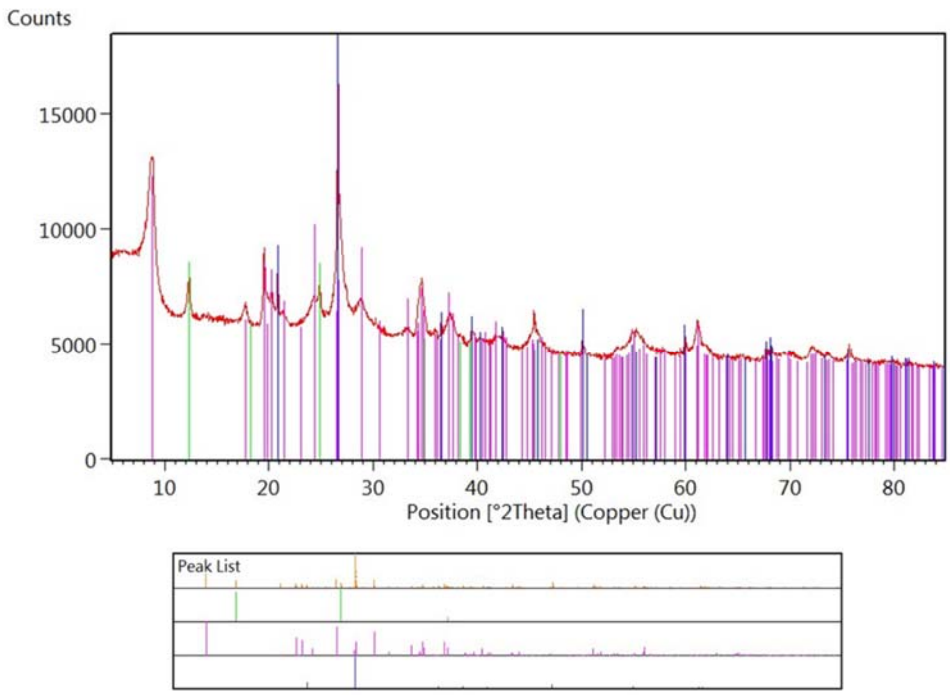

Figure S4. Identification of crystalline phases of glauconite clay. The presence of glauconite (pink), kaolinite (light green) and quartz (dark blue) had been proven using the ICDD PDF4+2021 diffraction database.

Table S1. The results of semiquantitative phase analysis by the Rietveld method.

Ref. Code	Compound Name	Chemical Formula	Mineral name	SemiQuant [%]
01-075-8322	Silicon Oxide	SiO_2	Quartz	14.5(4)
04-017-0528	Potassium Sodium Magnesium Aluminum Iron Silicon Oxide Hydroxide	$K_{0.84}Na_{0.01}Mg_{0.38}Fe_{1.22}Al_{0.71}Si_{3.72}O_{10}(OH)_2$	Glauconite	81(1)
00-058-2006	Aluminum Silicate Hydroxide	$Al_2Si_2O_5(OH)_4$	Kaolinite	5(1)
